# Transcriptome Analysis of Mesenchymal Progenitor Cells Revealed Molecular Insights into Metabolic Dysfunction and Inflammation in Polycystic Ovary Syndrome

**DOI:** 10.3390/ijms25147948

**Published:** 2024-07-20

**Authors:** Mei-Chi Huang, Pei-Lung Chen, Chia-Lang Hsu

**Affiliations:** 1 Graduate Institute of Medical Genomics and Proteomics, College of Medicine, National Taiwan University, Taipei 100, Taiwan; d00455003@ntu.edu.tw; 2 Department of Medical Genetics, National Taiwan University Hospital, Taipei 100, Taiwan; 3 Department of Medical Research, National Taiwan University Hospital, Taipei 100, Taiwan; 4 Graduate Institute of Oncology, National Taiwan University College of Medicine, Taipei 100, Taiwan

**Keywords:** polycystic ovary syndrome, induced pluripotent stem cells, RNA sequencing

## Abstract

Polycystic ovary syndrome (PCOS) is a female endocrine disorder with metabolic issues. Hyperandrogenism combined with hyperinsulinemia exacerbates the reproductive, metabolic, and inflammatory problems in PCOS patients. The etiology of PCOS is unclear. Patient-specific induced pluripotent stem cells (iPSCs) offer a promising model for studying disease mechanisms and conducting drug screening. Here, we aim to use mesenchymal progenitor cells (MPCs) derived from PCOS iPSCs to explore the mechanism of PCOS. We compared the transcriptome profiles of PCOS and healthy control (HC) iPSC-derived MPCs (iPSCMs). Moreover, we assess the impact of androgens on iPSCMs. In the comparison between PCOS and HC, the expression levels of 1026 genes were significantly different. A gene set enrichment analysis (GSEA) revealed that adipogenesis- and metabolism-related genes were downregulated, whereas inflammation-related genes were upregulated in the PCOS iPSCMs. Dysregulation of the TGF-β1 and Wnt signaling pathways was observed in the PCOS iPSCMs. Furthermore, there was impaired adipogenesis and decreased lipolysis in the PCOS iPSCMs-derived adipocytes. With testosterone treatment, genes related to metabolism were upregulated in the HC iPSCMs but downregulated in the PCOS iPSCMs. The impact of testosterone varied among HCs and PCOS iPSCMs, possibly because of a genetic predisposition toward PCOS. This study found specific signaling pathways that could serve as therapeutic targets for PCOS.

## 1. Introduction

Polycystic ovary syndrome (PCOS) is a common endocrine disorder affecting 5–10% of women of reproductive age. In 1935, Drs. Stein and Leventhal first described PCOS in obese women with acne, amenorrhea, hirsutism, and polycystic ovaries who were seeking help for infertility problems [1,2]. The 2003 ESRHE/ASRM Rotterdam consensus meeting proposed the diagnostic criteria for PCOS: (1) oligo- and/or anovulation, (2) clinical and/or biochemical signs of hyperandrogenism, and (3) polycystic ovaries; at least two of the three criteria are required for diagnosis [3]. The main characteristic of PCOS is hyperandrogenism, which impairs folliculogenesis and causes hormone imbalances. Most women with PCOS experience metabolic issues such as obesity and insulin resistance, making PCOS a significant public health concern. Therefore, women with PCOS are at greater risk of developing type II diabetes mellitus and cardiovascular disease [4,5]. When hyperandrogenism is combined with hyperinsulinemia, it worsens reproductive and metabolic dysfunction, along with inflammation in PCOS. PCOS is a multifactorial disease that involves a combination of different genes and environmental factors. Furthermore, gene–gene and gene–environment interactions increase the complexity of symptoms and consequences [6]. No single etiology can document the cause of PCOS. Thus, therapies are based on symptoms rather than targeting genes or specific mechanisms [2,7]. These treatments cannot cure PCOS but can alleviate the symptoms.

The causes of complex diseases involve many factors and affect many tissues. For example, the development of PCOS involves the hypothalamus-pituitary-ovary (HPO) axis [1] and other peripheral tissues [8,9]. Due to ethical concerns, researchers have only been able to analyze cells taken from the peripheral tissue of PCOS patients, not from their nervous system or internal organs. Additionally, the proliferation ability of somatic cells is limited, which restricts the assays via somatic cells. Hence, the definite mechanisms of PCOS have not been well established; one reason for the difficulties in studying PCOS is the lack of a suitable human disease model. Induced pluripotent stem cells (iPSCs) are a unique stem cell type derived by reprogramming somatic cells into embryonic stem cell (ESC)-like cells [10,11]. iPSCs can self-renew and produce cells characteristic of all three germ layers. Therefore, patient-specific iPSCs show great promise for establishing disease models to explore disease mechanisms, perform drug screening, and develop regenerative medicine approaches [12].

Using iPSCs derived from PCOS patients, we can obtain various cell types, such as neuron-related cells, muscle cells, and ovary-related cells, to study the mechanism of PCOS. In this study, we aimed to use iPSCs derived from PCOS patients to generate mesenchymal progenitor cells (MPCs). We explored the difference in transcriptome profiles between PCOS and healthy control (HC) iPSC-derived MPCs (iPSCMs). MPCs are equivalent to mesenchymal stem cells (MSCs), which are present throughout the human body and are involved in critical functions such as tissue and organ homeostasis, aging, and fibrosis [13]. They are progenitor cells that can differentiate into adipocytes, osteoblasts, and chondrocytes. In addition, MSCs can modulate the immune system by responding to proinflammatory signals and releasing proinflammatory/anti-inflammatory factors [14,15,16]. By using MPCs, we can find clues about the pathogenesis of PCOS. Moreover, we treated iPSCMs with testosterone to explore the effect of androgens on iPSCMs. From these results, we could gain a deeper understanding of PCOS and potentially identify therapeutic targets in patients with PCOS. Furthermore, PCOS iPSCs can differentiate into other cell lineages, which enables the exploration of whether abnormalities occur throughout the body or only in specific organs/cells.

## 2. Results

### 2.1. iPSC Establishment and MPC Derivation

**Figure 1:**
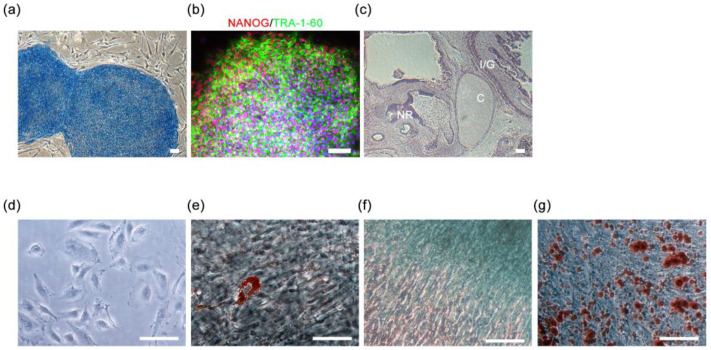
Characterization of induced pluripotent stem cells (iPSCs) and iPSC-derived mesenchymal progenitor cells (iPSCMs). (**a**) Alkaline phosphatase staining showing alkaline phosphatase activity in iPSCs (blue). (**b**) Immunostaining for the pluripotency markers NANOG (red) and TRA-1-60 (green) in iPSCs. NANOG was expressed in the nuclei, while TRA-1-60 was expressed on the cell surface. Nuclei were stained with Hoechst 33,342 (blue). (**c**) Hematoxylin and eosin (H&E) staining of teratomas derived from iPSCs. The markers show the sites of lineage-specific morphological characteristics, including neuroepithelial rosette (NR) for ectoderm, chondrocyte (C) for mesoderm, and intestinal or gut-like epithelium (I/E) for endoderm. (**d**) Bright-field image showing the fibroblast-like spindle morphology of iPSCMs. (**e**) Oil red O staining of lipid droplets in adipocytes derived from iPSCMs. (**f**) Alcian blue staining for acidic polysaccharides in chondrocytes derived from iPSCMs. (**g**) Alizarin Red S staining of calcium nodules in osteoblasts derived from iPSCMs. Scale bars, 100 mm.

To explore the mechanism underlying PCOS, we first aimed to derive MPCs from the iPSCs of PCOS patients. To generate PCOS iPSCs, we collected luteinized granulosa cells from PCOS patients who were undergoing in vitro fertilization, and cells from healthy individuals were included as controls. The donors presented at least two symptoms according to the Rotterdam diagnostic criteria, and their ages ranged from 33 to 39 years. To confirm the pluripotency of the obtained iPSCs, we evaluated the expression of pluripotency markers and their ability to produce cells belonging to three germ layers. The staining results showed that these iPSCs exhibited alkaline phosphatase activity (Figure 1a) and expressed the pluripotency markers NANOG and TRA-1-60 (Figure 1b). Teratoma formation assays confirmed the ability of these iPSCs to differentiate into three germ layers. The teratomas formed by the iPSCs displayed characteristics of three germ layers after H&E staining: neuroepithelial rosettes for the ectoderm, chondrocytes for the mesoderm, and intestinal or gut-like epithelium for the endoderm (Figure 1c). These results showed that the iPSCs derived from the patients’ granulosa cells were pluripotent and could be utilized to generate MPCs.

MPC induction was carried out using a mesenchymal progenitor kit. iPSC-derived MPCs (iPSCMs) exhibited a fibroblast-like spindle shape (Figure 1d). To determine whether these iPSCMs could generate three different cell types—adipocytes, osteoblasts, and chondrocytes—we induced their differentiation into specific lineages and confirmed their differentiation through staining with specific dyes. Oil Red O staining confirmed the presence of lipid droplets in adipocytes derived from iPSCMs (Figure 1e). The acidic polysaccharides in the iPSCM-derived chondrocytes were stained with Alcian blue (Figure 1f). Alizarin Red S staining showed that calcium nodules were formed in osteoblasts derived from iPSCMs (Figure 1g). These results indicated that these iPSCMs were similar to MSCs which could be utilized for transcriptome assays.

### 2.2. Transcriptome Differences between iPSCMs from PCOS Patients and Those from Healthy Controls

**Figure 2:**
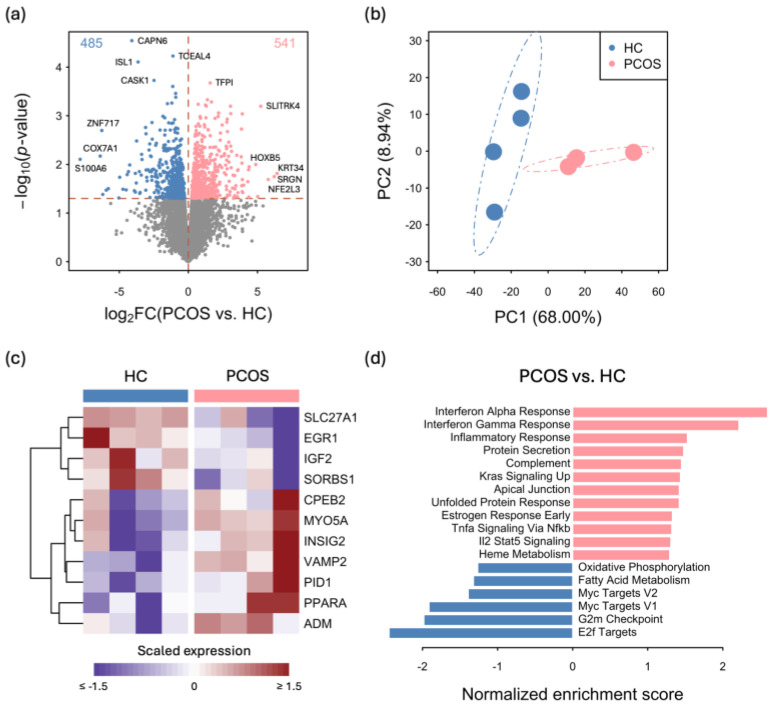
Transcriptomic analyses of PCOS and HC iPSCMs. (**a**) The volcano plot depicts the results of the differential expression analysis comparing PCOS and HC iPSCMs, highlighting the differentially expressed genes (DEGs), with red indicating upregulated genes and blue indicating downregulated genes. (**b**) Principal component analysis (PCA) using DEGs illustrating distinct expression profiles between PCOS and HC iPSCMs. (**c**) Expression of DEGs related to the insulin response across all samples. (**d**) The bar plot displays the significantly enriched hallmark gene sets. All gene sets with *p* < 0.05 were derived from a gene set enrichment analysis (GSEA). HC: healthy control.

To investigate the functional differences between the iPSCMs from PCOS patients and those from healthy controls (HCs), an RNA-seq analysis was conducted on eight iPSCMs, four from individuals with PCOS and four from HCs. Compared with those in HCs, 541 genes were upregulated, while 485 genes were downregulated in the PCOS iPSCMs (Figure 2a and Table S1). A principal component analysis (PCA) of the expression profiles of these 1026 differentially expressed genes revealed a distinct separation between the PCOS and HC iPSCMs along PC1 (Figure 2b). This indicated robust differences in gene expression patterns between the two groups, suggesting that unique molecular signatures are associated with the pathophysiology of PCOS. The expression of several genes involved in insulin regulation, which has been proposed as an etiological factor of PCOS, differed between the PCOS and HC iPSCMs (Figure 2c). For example, SLC27A1 is an insulin-sensitive fatty acid transporter and abnormal SLC27A1 expression may lead to insulin resistance [17]. *SLC27A1* expression was increased in the HC iPSCMs but decreased in the PCOS iPSCMs (Figure 2c). A gene set enrichment analysis (GSEA) of the hallmark gene sets from MSigDB revealed differences in various functions between the PCOS and HC iPSCMs (Figure 2d). Compared with those in the HC iPSCMs, genes that respond to estrogen were found to be differentially regulated in the PCOS iPSCMs, indicating an abnormal hormone response in PCOS patients. The levels of the G2M checkpoint and E2F targets were lower in the PCOS iPSCMs than in HCs, suggesting dysregulation of the proliferation of PCOS iPSCMs. Moreover, genes associated with the metabolism and the immune response were differentially expressed between the PCOS and HC iPSCMs, and we further analyzed these genes and their signatures.

### 2.3. The Expression Levels of Adipogenesis- and Metabolism-Related Genes Were Decreased in PCOS iPSCMs

**Figure 3:**
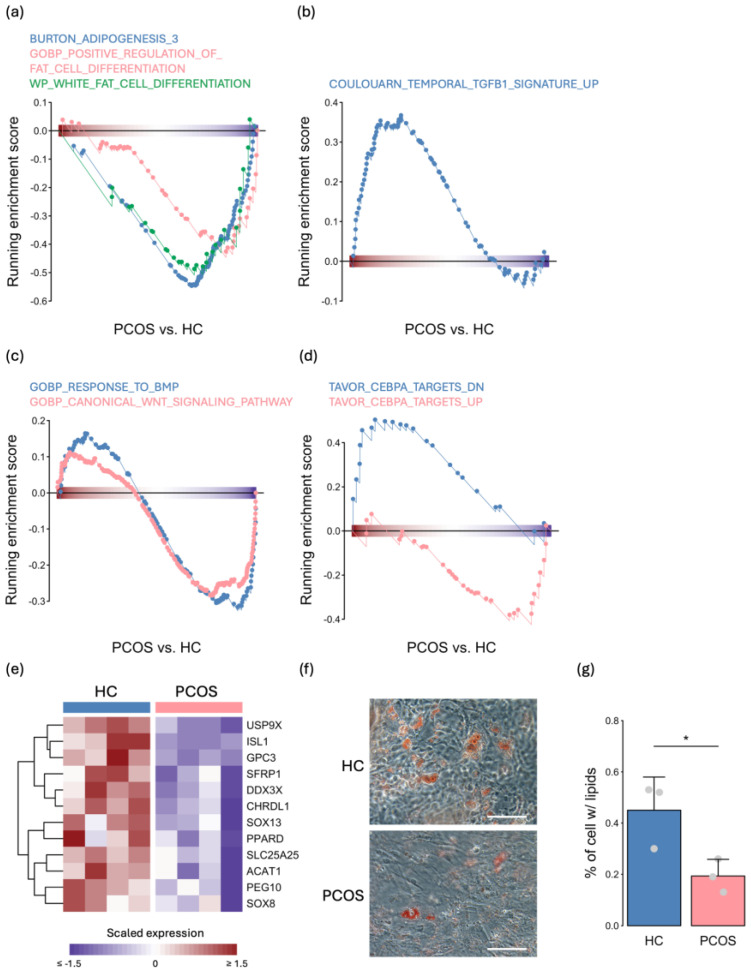
Transcriptomic analysis revealed adipogenesis dysfunction in PCOS iPSCMs. (**a**–**d**) GSEA plots of gene sets related to adipogenesis-related pathways (**a**), TGF-β1-related pathways (**b**), BMP- and Wnt-related pathways (**c**), and C/EBPA-related pathways (**d**). *p* < 0.05 for all gene sets. (**e**) Expression of genes related to adipogenesis across all samples. (**f**) Oil red O staining showing iPSCM-derived adipocytes with lipids. Scale bars, 100 μm. (**g**) Flow cytometry quantification of iPSCM-derived adipocytes with lipid droplets. The experiments were conducted in biological triplicate. Error bar represents the mean ± SD. *t*-test: * *p* < 0.05. HC: healthy control.

The hallmark gene set analysis revealed a significant decrease in the expression of genes associated with cell proliferation and fatty acid metabolism in the PCOS iPSCMs (Figure 2d). This finding suggests a potential dysfunction in the differentiation process of fat cells. Indeed, compared to those from the HCs, PCOS iPSCMs exhibited negative enrichment of genes related to fat cell differentiation and adipogenesis (Figure 3a). Furthermore, the upregulation of signatures associated with TGF-β1, which inhibits MPC commitment to preadipocytes [18], was observed in the PCOS iPSCMs (Figure 3b). In contrast, downregulation of the Wnt signaling pathway, a key switch in adipogenesis [18], and the BMP signaling pathway, which triggers MPC commitment to adipocytes [18], was observed in the PCOS iPSCMs compared to the HC iPSCMs (Figure 3c). Additionally, the GSEA revealed that the transcriptional activity of C/EBPA, a crucial transcription factor for adipogenesis [18], was suppressed in the PCOS iPSCMs (Figure 3d). The downregulation of genes involved in the Wnt signaling pathway, such as *GPC3* and *DDX3X*, along with *CHRDL1* and *SFRP1*, which are implicated in the BMP signaling pathway, was also observed in the PCOS iPSCMs (Figure 3e). To validate the adipogenic potential of these iPSCMs, we subjected them to adipogenic induction. Oil Red O staining revealed fewer cells containing lipid droplets among PCOS iPSCM-derived adipocytes (Figure 3f). Consistent results were obtained through the quantification of cells containing lipid droplets using a fluorescent lipid dye (*p* < 0.05) (Figure 3g). Based on these results, the factors involved in adipogenesis differed between the PCOS and HC iPSCMs. In addition, the adipogenesis efficiency was decreased in the PCOS iPSCMs.

**Figure 4:**
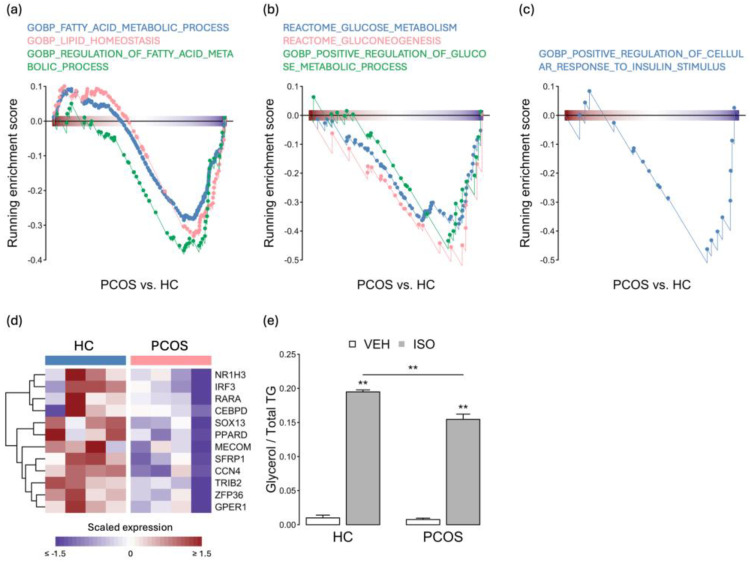
The suppression of fatty acid and glucose metabolism pathways in PCOS iPSCMs. (**a**–**c**) GSEA plots reveal the expression of gene sets associated with fatty acid metabolism (**a**), glucose metabolism (**b**), and the cellular response to insulin stimulation pathways (**c**) in iPSCMs from individuals with PCOS compared to those from the HCs. *p* < 0.05 for all gene sets. (**d**) Heatmap illustrating the expression of genes related to metabolic pathways across all samples. (**e**) Isoproterenol-induced glycerol release normalized to triglyceride levels in iPSCM-derived adipocytes. The experiments were conducted in biological triplicate. Error bar represents the mean ± SD. *t*-test: ** *p* < 0.01. TG: triglyceride, VEH: vehicle, ISO: isoproterenol, HC: healthy control.

Impaired adipogenesis can lead to dysfunction in adipose tissue, ultimately resulting in metabolic issues. A transcriptome analysis revealed a decrease in the expression of genes involved in the fatty acid metabolism in the PCOS iPSCMs (Figure 4a). Furthermore, a downregulated expression of genes related to glucose metabolism was observed in the PCOS iPSCMs (Figure 4b). Additionally, genes involved in the cellular response to insulin stimulation, which is crucial for GLUT4 translocation to facilitate glucose uptake, tended to be downregulated in the PCOS iPSCMs (Figure 4c). Moreover, genes associated with glucose homeostasis (such as *PDFK* and *IGF2*) and fatty acid homeostasis (such as *LIPG* and *PDK*) were expressed at low levels in the PCOS iPSCMs compared to the HCs (Figure 4d). Lipolysis is the process through which adipocytes break down triglycerides into fatty acids and glycerol. After adipogenic induction, we observed a significant reduction in lipolysis induced by isoproterenol in adipocytes generated from the PCOS iPSCMs (*p* < 0.01) (Figure 4e). These findings suggest that adipocyte development and metabolic processes were impaired in the PCOS iPSCMs.

### 2.4. Activation of the Immune Response in PCOS iPSCMs

**Figure 5:**
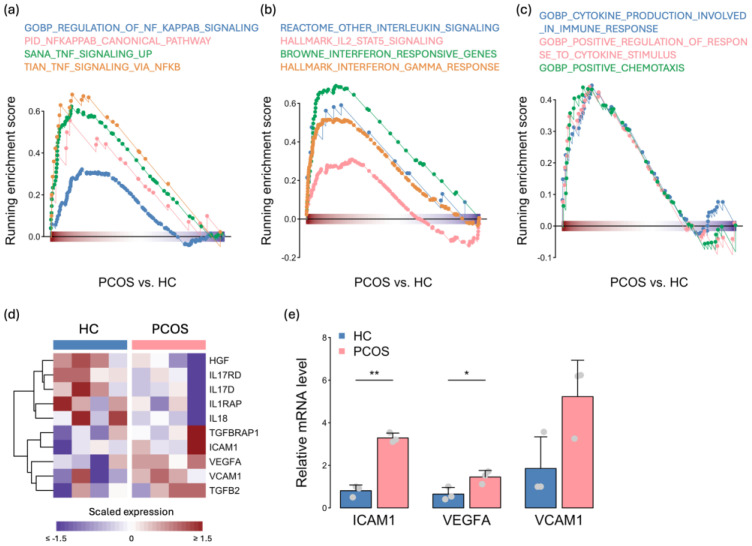
The increase in the expression of immune-related genes in PCOS iPSCMs. (**a**–**c**) GSEA plots illustrating the expression of gene sets associated with TNF and NFκB signaling (**a**), interleukin and interferon responses (**b**), and cytokine and chemotaxis elements between iPSCMs from PCOS patients and HCs (**c**). *p* < 0.05 for all gene sets. (**d**) Relative expression of cytokines across all samples. (**e**) *ICAM1, VEGFA,* and *VCAM1* expression in the granulosa cells were analyzed via real-time quantitative PCR. One control sample was used for normalization. The experiments were conducted in biological triplicate. Error bar represents the mean ± SD. *t*-test: * *p* < 0.05, ** *p* < 0.01. HC: healthy control.

In addition to metabolism, inflammation response signatures were highly enriched according to transcriptome profiling, and we further investigated immune-related gene sets (Figure 2d). MPCs can respond to proinflammatory signals and release both proinflammatory and anti-inflammatory factors to regulate the immune system. The expression of genes related to TNF and NFκB signaling, which regulate several biological processes, was greater in the PCOS iPSCMs than in the HC iPSCMs (Figure 5a). The expression levels of other inflammatory response genes, such as interleukins and interferons, were also elevated in the PCOS iPSCMs (Figure 5b). In addition, the expression of genes involved in cytokine and chemotaxis release was found to be upregulated in the PCOS iPSCMs compared to the HC iPSCMs (Figure 5c). The expression of cytokines secreted by the MPCs differed between the PCOS and HC iPSCMs (Figure 5d), and increased ICAM1 and VCAM1 have been reported in PCOS patients [19,20]. Furthermore, the expression of these cytokines was confirmed in the granulosa cells from patients by real-time quantitative PCR, and the results showed *ICAM1* and *VEGFA* were higher in PCOS patients than in the controls (*p* < 0.05) (Figure 5e). These results revealed abnormalities in cytokine production and the immune response in iPSCMs from individuals with PCOS.

### 2.5. Distinct Effects of Testosterone Treatment on PCOS and HC iPSCMs

**Figure 6:**
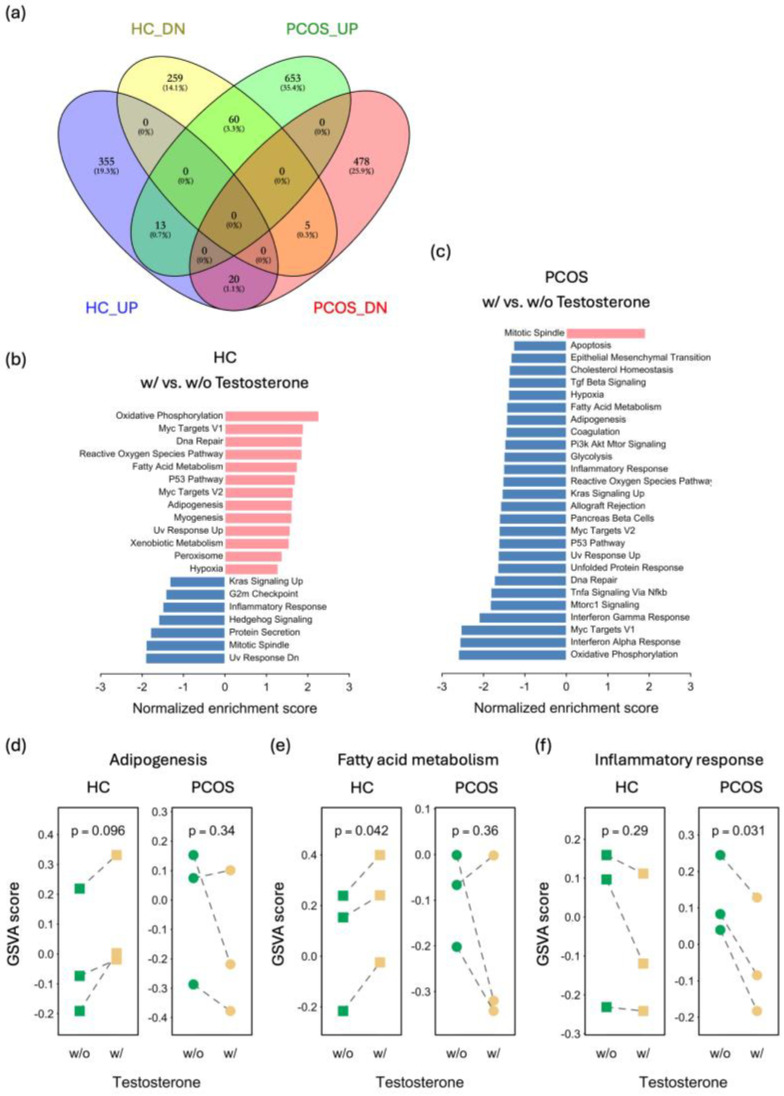
Transcriptomic analysis revealing the impact of testosterone on iPSCMs from PCOS patients and HCs. (**a**) Venn diagram depicting differentially expressed genes (DEGs) in PCOS and HC iPSCMs with and without testosterone treatment. (**b**,**c**) The bar plot displays the significantly enriched hallmark gene sets in HC iPSCMs with vs. without testosterone treatment (**b**) and in PCOS iPSCMs with vs. without testosterone treatment (**c**). All gene sets with *p* < 0.05 were derived from the GSEA. (**d**,**e**) Gene set variation analysis (GSVA) showing the activity of hallmark gene sets related to adipogenesis (**d**), fatty acid metabolism (**e**), and the inflammatory response (**f**) for each sample. Green: cells without testosterone treatment, jasmine: cells with testosterone treatment, squares: healthy control, circles: PCOS patient. The *p* values were derived from a paired *t*-test. HC_DN: genes whose expression was downregulated in HC iPSCMs with testosterone treatment vs. those without testosterone treatment, HC_UP: genes whose expression was upregulated in HC iPSCMs treated with testosterone vs. those without testosterone, PCOS_DN: genes whose expression was downregulated in PCOS iPSCMs with vs. without testosterone treatment, PCOS_UP: genes whose expression was upregulated in PCOS iPSCMs with vs. without testosterone treatment, HC: healthy control, w/o: without, w/: with.

Most patients with PCOS exhibit hyperandrogenism, which is crucial in the development of PCOS. To investigate the effect of testosterone on MPCs, a transcriptome analysis was conducted on iPSCMs treated with testosterone. Due to poor data quality, two clones (one from the PCOS group and one from the HC group) were excluded from further analysis. When comparing iPSCMs with and without testosterone treatment, 712 DEGs were identified in the HC iPSCMs, whereas 1229 DEGs were identified in the PCOS iPSCMs. Interestingly, only 97 genes were commonly identified in both the PCOS and HC iPSCMs (Figure 6a). Among these 97 genes, 79 showed opposite expression trends in the response to testosterone, suggesting a distinct effect of testosterone. Similarly, the GSEA of the hallmark gene sets revealed significant differences in functional pathways following testosterone treatment between the PCOS and HC iPSCMs (Figure 6b,c). For instance, genes associated with oxidative phosphorylation, the reactive oxygen species pathway, adipogenesis, and fatty acid metabolism were upregulated in the HC iPSCMs but downregulated in the PCOS iPSCMs upon testosterone treatment (Figure 6b,c). Nevertheless, the expression of genes related to the inflammatory response was reduced in both the PCOS and HC iPSCMs after testosterone treatment (Figure 6b,c).

To further explore the effects on individual clones, we utilized the gene set variation analysis (GSVA) method to assess the enrichment level of gene sets and pathways in each sample. Due to the limited sample size and individual variability, the observed differences did not reach statistical significance; however, each clone displayed a similar trend following testosterone treatment. According to the above results, impaired fat cell differentiation and adipogenesis were observed in the PCOS iPSCMs. Following testosterone treatment, genes associated with adipogenesis and fatty acid metabolism remained downregulated in the PCOS iPSCMs but were upregulated in the HC iPSCMs (Figure 6d,e). Additionally, the expression of genes related to the inflammatory response was downregulated in response to testosterone in both the PCOS and HC iPSCMs (Figure 6f). These results indicate that testosterone has very different effects on adipogenesis and fatty acid metabolism but have the same effect on the inflammatory response in the PCOS and HC iPSCMs.

## 3. Discussion

In this study, we aimed to explore the underlying mechanism of PCOS by using PCOS iPSCMs. To achieve this goal, we performed RNA sequencing to compare the transcriptomes of PCOS and HC iPSCMs. We discovered differences in the expression of genes related to adipogenesis, metabolic function, and the immune response between the PCOS and HC iPSCMs. The signaling pathways that inhibited early adipogenesis, such as the TGF-β1 signaling pathway, were upregulated, while those that promoted adipogenesis, including the Wnt, BMP, and C/EBPA signaling pathways, were downregulated. The genes associated with fatty acid and glucose metabolism were downregulated in the PCOS iPSCMs. The inflammatory response and cytokine release were increased in the PCOS iPSCMs. In addition, we studied the impact of testosterone on iPSCMs. The results showed that testosterone may promote adipogenesis and fatty acid metabolism in HC iPSCMs but may have a detrimental effect on the PCOS iPSCMs. Moreover, testosterone inhibited the inflammatory response in both the PCOS and HC iPSCMs.

Both nonobese and obese women with PCOS can have abdominal obesity, which is linked to a more harmful metabolic profile. When energy intake exceeds energy expenditure, fat storage increases via the enlargement of mature adipocytes (hypertrophy) and the generation of new adipocytes (hyperplasia). Impaired adipogenesis or dysfunction in fat storage causes hypertrophy, thus resulting in ectopic lipid accumulation and lipotoxicity. Some studies have found enlarged adipocytes in PCOS patients [21,22], whereas others have observed an increase in small adipocytes in PCOS patients [23]; the latter suggested a limited capacity of these small adipocytes to store fat. The mechanism by which impaired adipogenesis leads to metabolic dysfunction in PCOS patients has not been fully explored, and most findings were obtained from cells/animals treated with androgen. Chazenbalk et al. revealed dysregulated Wnt signaling in the adipose tissue of women with PCOS [24]. Decreased *C/EBPA* levels were found in the adipose tissue of androgen-treated monkeys [25], and C/EBPα and PPARγ were downregulated in androgen-treated cells [26,27,28]. MPCs are at an earlier developmental stage than adipocytes, and we could explore the mechanism of early adipogenesis by using MPCs. The TGF-β, BMP, and Wnt signaling pathways are responsible for MSC commitment to the preadipocytes, while C/EBPA and PPARG assist in terminal differentiation to form mature adipocytes [18,29]. Various adipogenesis-related signaling pathways were affected in our PCOS iPSCMs. Because the TGF-β1, BMP, and Wnt signaling pathways are activated earlier in adipogenesis than the C/EBPA and PPARG pathways, we suggest that impaired adipogenesis may be caused by dysregulation of the TGF-β1, BMP, and Wnt signaling pathways. However, the primary signaling pathway that causes impaired adipogenesis in PCOS patients needs further exploration.

**Figure 7:**
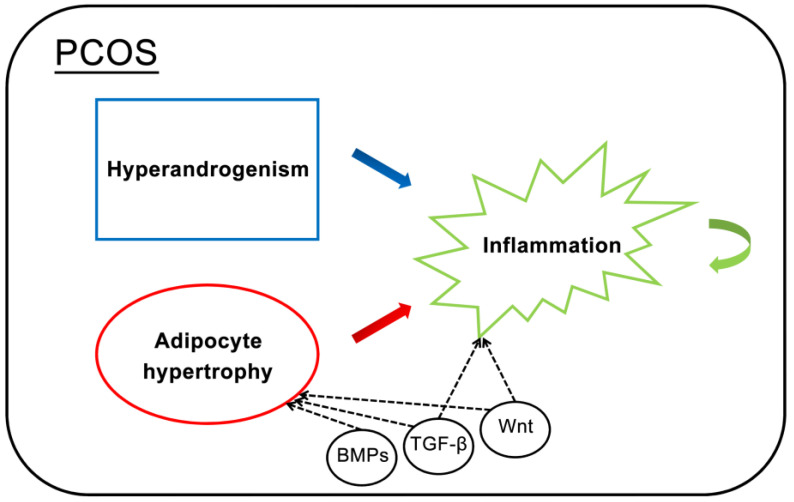
The causes of inflammation in PCOS. Both hyperandrogenism and adipocyte hypertrophy contribute to inflammation in patients with PCOS. However, inflammation can occur due to dysregulation in inflammatory genes independent of hyperandrogenism and adipocyte hypertrophy. Dysregulation in BMPs, TGF-β, and Wnt may be involved in adipocyte hypertrophy and inflammation in polycystic ovary syndrome (PCOS), as observed in PCOS iPSCMs.

Increased expression of inflammatory markers such as C-reactive protein (CRP), interleukins, and oxidative stress factors in PCOS contributes to the development of chronic inflammation [30]. The general view in reports is that impaired adipogenesis or fat storage dysfunction causes hypertrophy, which occurs with hypoxia, leading to increased inflammation [31]. Most studies propose that inflammation in PCOS patients is often due to obesity and hyperandrogenism, but there is some evidence showing abnormal inflammatory cytokine levels in the ovarian cells of PCOS patients [32]. Piltonen et al. reported the dysregulation of inflammatory genes in the endometrial cells of PCOS patients [33]. A meta-analysis revealed that women with PCOS exhibit an increase in circulating CRP that is independent of obesity [34]. Without additional androgen treatment and adipocyte hypertrophy, there were differences in cytokine release and the immune response between the PCOS and HC iPSCMs. This result indicates that the dysregulation of inflammatory genes directly causes inflammation in PCOS patients, regardless of obesity or hyperandrogenism. Abnormal expression of these factors in the PCOS iPSCMs was also found in the granulosa cells of PCOS patients, which are the parent cells used to derive iPSCs. Furthermore, inflammatory genes were dysregulated in both somatic cells and the cells after somatic cell reprogramming, showing genetic defects in inflammatory genes in our PCOS patients. Some factors are multifunctional and can regulate various biological processes. Dysregulation of these factors can lead to the dysfunction of multiple biological functions. For example, TGF-β regulates adipogenesis, glycolysis, and inflammatory activity. The rs4803457C/T polymorphism in the TGF-β1 gene is correlated with susceptibility to PCOS and contributes to PCOS development in Chinese women [35]. It can be secreted by MPCs, and genes related to the TGF-β1 signaling pathway differ between the PCOS and HC iPSCMs. Another example is Wnt5a, a proinflammatory marker associated with obesity [36], which has been reported to be increased in granulosa cells of PCOS patients [37]. Wnt5a can inhibit adipogenesis [18] and is upregulated in the PCOS iPSCMs. These molecules may contribute to both metabolic dysfunction and chronic inflammation in PCOS patients. The causal relationship between hyperandrogenism, adipocyte hypertrophy, and inflammation, along with the molecules related to these conditions in PCOS, is illustrated in Figure 7.

Hyperandrogenism impairs folliculogenesis and causes hormone imbalances in more than 60–80% of PCOS patients [7]. Androgen treatment impairs adipogenesis and causes metabolic dysfunction in both animal models [25] and cell culture systems [26]. In this study, testosterone treatment was conducted for three weeks. Metabolism-related pathways, such as adipogenesis and fatty acid metabolism, were upregulated in the HC iPSCMs but downregulated in the PCOS iPSCMs. The response to testosterone differed between the HCs and individuals with PCOS, which may be due to a genetic predisposition to PCOS. In another study performed by our group, testosterone treatment was carried out for two months during adipogenesis. Both the HC and PCOS iPSCM-derived adipocytes were impaired. We speculate that short-term androgen stimulation may benefit metabolic functions in HCs, such as adipogenesis and fatty acid metabolism, which are upregulated in the HC iPSCMs. However, long-term hormone imbalance may lead to dysfunction of tissues and organs, such as adipose tissue. Generally, androgens have anti-inflammatory effects and can suppress immune cell activity [38], but the anti-inflammatory effects of androgens are not consistent. Hyperandrogenism in PCOS may change inflammation by affecting immune cells [39]. Short-term testosterone treatment in our model decreased the inflammatory response in both the PCOS and HC iPSCMs. For long-term androgen treatment, the effect of testosterone on iPSCMs needs to be confirmed.

Patient-specific iPSCs could serve as an inexhaustible source of cells with genetic information specific to the patient and reflective of their pathogenesis. Various lineages of cells can be derived, and we can utilize these cells to explore the underlying mechanism through various assays, such as gene expression and functional assays. The limitations of the iPSC model include the heterogeneity of cell differentiation and the inability to consider tissue crosstalk. The development of purification methods to increase cell homogeneity is important. By sorting with a specific surface marker, we can either include or exclude a subpopulation from the total cell population. Another approach is to use a serum-free, chemically defined medium for differentiation to minimize the complexity arising from undefined components. To address the issue of tissue crosstalk, co-culturing with other cells can resolve it. There are several methods to achieve the goal, including using transwell inserts and overflow culture chambers for indirect contact, as well as controlling the seeding positions of adherent cells for direct contact [40,41]. Indirect contact allows receiving secretory factors from other cells, which is suitable for studying complex endocrine disorders like PCOS. However, results from different models or platforms need to be integrated to better understand the underlying mechanism involved in these effects.

PCOS is implicated in multiple hormone interactions. In addition to testosterone, insulin is a vital factor in the development of PCOS. Hyperinsulinemia can promote free androgen production and cause insulin resistance [2]. In the future, we can evaluate the effect of insulin on the iPSCMs, combined with manipulating the crucial factors found in our study. Thus, we can gain a better understanding of the detailed molecular mechanism of PCOS. Furthermore, we can examine these iPSCMs with candidate chemicals or drugs to identify potential treatment options for PCOS. There are many tissues involved in the development of PCOS, including the hypothalamus-pituitary-ovary (HPO) axis and other peripheral tissues. Finally, we can differentiate these iPSCs into other cell lineages or co-culture them with different cell types to obtain a thorough insight into PCOS.

## 4. Materials and Methods

### 4.1. iPSC Derivation and Maintenance

Follicular aspirates were collected during oocyte retrieval from both healthy controls and PCOS patients who were undergoing in vitro fertilization for infertility treatment with GnRH antagonist and hCG. Granulosa cells in the follicular aspirates were isolated by using Ficoll density gradient centrifugation. The cells were cultured in an RPMI 1640 medium supplemented with 15% fetal bovine serum, 1% MEM nonessential amino acids (NEAA), 1% GlutaMAX, and 1% antibiotic-antimycotic. The virus infection and iPSC induction procedures followed the manufacturer’s instructions (Invitrogen, Waltham, MA, USA). In brief, granulosa cells were seeded at 2–5 × 10^5^ cells per 35 mm dish two days before infection. The cells were infected with either the first-generation Sendai virus at an MOI of 6 or the second-generation Sendai virus at an MOI of 5:5:3 (KOS:hc-Myc:hKlf4). Twenty-four hours after infection, the virus-containing medium was removed, and the medium was replaced with fresh medium daily. One week after infection, the cells were collected using Accumax (eBioscience, San Diego, CA, USA) and replated on mitomycin C-inactivated mouse (BALB/c) embryo fibroblasts as feeder cells. The next day, the medium was changed to DMEM/F12 supplemented with 20% knockout serum replacement, 1% NEAA, 0.5% GlutaMAX, 1% penicillin–streptomycin, and 5 ng/mL basic fibroblast growth factor (PeproTech, Cranbury, NJ, USA). After one month of infection, the colonies were hand-picked and placed on feeder cells. The iPSCs were manually passaged weekly, and the medium was replaced daily. When iPSCs were cultured without feeders, they were maintained in Essential 8 medium on vitronectin-coated dishes. These cells were passaged using 0.5% EDTA in Dulbecco’s phosphate-buffered saline (DPBS) every 5 days, and the medium was changed daily. Cell culture media, serum, and buffers were procured from Life Technologies (Grand Island, NY, USA), and the chemicals were procured from Sigma-Aldrich (St. Louis, MO, USA) unless otherwise stated.

### 4.2. MPC Differentiation and Testosterone Treatment

The STEMdiff mesenchymal progenitor kit (STEMCELL, Vancouver, BC, Canada) was utilized to differentiate MPCs. Two days before induction, iPSCs were cultured without feeder cells and treated with 10 μM Y-27632 (Merck, Darmstadt, Germany) for 2 h. Then, the iPSCs were dissociated into single cells using TypLE and replated at a concentration of 5 × 10^4^ cells/cm^2^ in Essential 8 medium supplemented with 10 μM Y-27632. The next day, the medium was replaced with fresh medium. From the start of day 0 until day 3, the medium was changed to a mesenchymal induction medium. On day 4, the medium was changed to a MesenClut-ACF medium. On day 6, the cells were treated with 10 μM Y-27632 for 2 h. Then, the cells were dissociated using TypLE and replated at a concentration of 3–10 × 10^3^ cells/cm^2^ in a MesenClut-ACF medium on MesenClut-ACF attachment substrate-coated plates. Once the cells reached 80% confluence, 10^−7^ M of testosterone was added for three weeks, and the medium was changed weekly.

### 4.3. Teratoma Formation

iPSCs (1–3 × 10^7^) were treated with 10 μM Y-27632 for 2 h and dissociated into single cells by using TypLE. The cells were suspended in DPBS and subcutaneously injected into the dorsal flank of a NOD/SCID mouse. Two months after injection, the formed tumors were dissected and fixed with 4% paraformaldehyde (PFA) in phosphate-buffered saline (PBS). Paraffin-embedded tissue was sliced and stained with hematoxylin and eosin.

### 4.4. Staining for Alkaline Phosphatase Activity and Immunocytochemistry

Alkaline phosphatase activity staining was carried out using an alkaline phosphatase substrate kit from Vector (Newark, CA, USA). In brief, the cells were incubated with the substrate working solution for 30 min and washed with PBS three times. Immunocytochemistry was performed by fixing iPSCs with 4% PFA in PBS for 30 min. Following washing with PBS, the cells were permeabilized with 0.2% Triton X-100 in 0.05% Tween 20/PBS for 30 min. The samples were blocked by incubation in 0.05% Tween 20 in PBS containing 3% BSA and 0.3 M glycine for 1 h. Then, the samples were incubated with a primary antibody (1:100 dilution with blocking buffer) overnight at 4 °C. After incubation with the primary antibody, the samples were washed with PBS three times and incubated with a secondary antibody (1:1000 dilution with blocking buffer) for 1 h at room temperature. Finally, the samples were washed with PBS three times and incubated with Hoechst 33,342 for 10 min at room temperature. Afterward, the samples were washed with PBS three times and preserved with a mounting medium (Bio-Rad, Hercules, CA, USA). This experiment involved the use of primary antibodies against NANOG (Cell Signaling, Danvers, MA, USA) and TRA-1-60 (Millipore, Burlington, MA, USA). Alexa Fluor-conjugated secondary antibodies were acquired from Jackson ImmunoResearch (West Grove, PA, USA).

### 4.5. Induction of Specific Lineages (Adipocytes, Osteoblasts, and Chondrocytes), Oil Red O Staining, Alizarin Red S Staining, and Alcian Blue Staining

Adipogenic, osteogenic, and chondrogenic differentiation were performed according to the manufacturer’s instructions (STEMCELL, Vancouver, BC, Canada). Adipogenic induction was performed when the iPSCMs reached 90% confluence, and the medium was changed to an adipogenic induction medium. The medium was changed every 5 days for one month. For Oil Red O staining, iPSCM-derived adipocytes were fixed with 4% PFA in PBS for 1 h. The cells were sequentially washed with PBS and 60% isopropanol. Then, the cells were stained with 0.3% Oil Red O in 60% isopropanol for 15 min and washed with distilled water four times. Osteogenic induction was carried out when the iPSCMs reached 90% confluence, and the medium was changed to an osteogenic induction medium. The medium was changed every 4 days until bone matrix formation occurred. For Alizarin Red S staining, iPSCM-derived osteoblasts were fixed with 4% PFA in PBS for 1 h. The cells were washed with distilled water twice and stained with an Alizarin Red S solution for 45 min. Then, the cells were washed with distilled water four times to remove unbound dye. A total of 1.25 × 10^7^ iPSCMs were cultured with 0.5 mL of chondrogenic induction medium in 15 mL polypropylene tubes (with the cap loosened) for chondrogenic differentiation. The medium was changed every 4 days for 21 days. iPSCM-derived cartilage spheroids were replated in wells coated with Matrigel (BD Biosciences, Franklin Lakes, NJ, USA) for 1 week. The cells were fixed with 4% PFA in PBS for 30 min and washed with PBS. The cells were stained with an Alcian blue solution for 1 h and sequentially washed with 0.1 M HCl and PBS twice. Image acquisition was performed using a Leica DM IRB digital microscope camera and NIS-Elements software (v3.22.14).

### 4.6. RNA Sequencing and Data Analysis

Total cell RNA was extracted using QIAzol Lysis Reagent (QIAGEN, Hilden, Germany). Libraries for RNA sequencing were prepared using the Illumina Stranded mRNA Prep Kit (San Diego, CA, USA) following the manufacturer’s protocol and subsequently sequenced on the Illumina NovaSeq600 platform (San Diego, CA, USA). The quality of the raw reads was assessed using FastQC (v0.11.9). Adaptor sequence trimming and poor read removal were performed using Cutadapt (v3.5). The qualified reads were aligned to the human reference genome GRCh38 using STAR (v2.7.8a) in two-pass mode. Gene counts were quantified based on the annotation in Gencode (v35) and converted into transcripts per million (TPM) to normalize for differences in library size and gene length. Genes with average expression values < 1 TPM were filtered out to focus on more reliably expressed genes. Between-sample normalization was conducted using the trimmed mean of M-values (TMM) method to account for compositional differences between samples and reduce potential batch effects. Differential expression analysis was performed via the R package limma. Batch effects and potential confounders were included in the model as covariates to improve the accuracy of differential expression calls. An overrepresentation analysis and gene set enrichment analysis (GSEA) of the gene sets from MSigDB were conducted using functions implemented in clusterProfiler. A single-sample-based enrichment analysis was performed via a gene set variation analysis (GSVA).

### 4.7. Flow Cytometry Analysis

To quantify the percentage of cells with lipid droplets, iPSCM-derived adipocytes were washed with DPBS and incubated with BODIPY 493/503 (Invitrogen, Waltham, MA, USA) in DPBS for 15 min. After washing with DPBS, these cells were dissociated into single cells by using TypLE and neutralized with PBS containing 2% FBS, 1 mM EDTA, 25 mM HEPES, and 2 µg/mL propidium iodide (PI). Quantification was performed using an Attune NxT flow cytometer (Thermo Fisher Scientific, Waltham, MA, USA).

### 4.8. Lipolysis Assay and Triglyceride Quantification

For the lipolysis assay, iPSCM-derived adipocytes were washed with DPBS and starved in DMEM containing 1% fatty acid-free BSA for 1 h. Then, the cells were stimulated with 100 nM isoproterenol in EBSS containing a 2% fatty acid-free BSA for 4 h. The supernatant was collected for subsequent measurement of glycerol concentrations (an index of lipolysis) by using a free glycerol reagent (Sigma-Aldrich, St. Louis, MO, USA). iPSCM-derived adipocytes were lysed and homogenized in a 5% NP-40 solution for triglyceride quantification. The samples were heated to approximately 80–100 °C and then cooled to room temperature twice. The samples were centrifuged, and the supernatant was collected to measure triglyceride levels using a serum triglyceride determination kit (Sigma-Aldrich). Lipolysis efficiency was determined according to the glycerol concentration normalized to the triglyceride concentration.

### 4.9. Real-Time Quantitative PCR

RNA (2 μg) was reverse transcribed using a cDNA synthesis kit with dsDNase (Thermo Fisher Scientific, Waltham, MA, USA). Quantitative PCR was performed using OMICS Green Master Mix (Omics Bio, New Taipei City, Taiwan), and the results were analyzed with a StepOnePlus System (Applied Biosystems, Waltham, MA, USA). The sequences of the utilized primers are VCAM1_forward: TTTGACAGGCTGGAGATAGACT, VCAM1_reverse: TCAATGTGTAATTTAGCTCGGCA, VEGFA_forward: AGGGCAGAATCATCACGAAGT, VEGFA_reverse: AGGGTCTCGATTGGATGGCA, ICAM1_forward: TTGGGCATAGAGACCCCGTT, ICAM1_reverse: GCACATTGCTCAGTTCATACACC, HPRT1_forward: TGACACTGGCAAAACAATGCA, and HPRT1_reverse: GGTCCTTTTCACCAGCAAGCT.

### 4.10. Statistical Analysis

Statistical analysis and data visualization for RNA-seq were conducted using the R language (v4.2.0). Other experimental data are shown as the mean ± SD. Statistical analysis was performed using PASW Statistics 18 and GraphPad Prism 6. *p* values were calculated using an independent sample *t*-test in SPSS to assess differences between the PCOS and HC iPSCM-derived adipocytes and paired sample *t*-tests for comparisons of untreated versus treated cells. The significance level was set at *p* < 0.05.

## 5. Conclusions

Hyperandrogenism, inflammation, and insulin resistance interact to create a harmful cycle in women with PCOS. This study revealed that several multifunctional factors, such as TGF-β1 and Wnts, are dysregulated in the PCOS iPSCMs. These factors may be involved in impaired adipogenesis, metabolic dysfunction, and immune system dysregulation. The next step is to explore the detailed action of these factors and their target pathways in the metabolic changes and immune activation observed in PCOS. Furthermore, we can screen drugs to improve metabolic issues and inflammation in PCOS patients.

## Data Availability

The original data presented in the study are openly available in Gene Expression Omnibus (GEO) at accession number GSE267287.

## Supplementary Materials

The following supporting information can be downloaded at: https://www.mdpi.com/article/10.3390/ijms25147948/s1.
